# Drug exposure in register-based research—An expert-opinion based evaluation of methods

**DOI:** 10.1371/journal.pone.0184070

**Published:** 2017-09-08

**Authors:** Antti Tanskanen, Heidi Taipale, Marjaana Koponen, Anna-Maija Tolppanen, Sirpa Hartikainen, Riitta Ahonen, Jari Tiihonen

**Affiliations:** 1 Karolinska Institutet, Department of Clinical Neuroscience, Stockholm, Sweden; 2 National Institute for Health and Welfare, Helsinki, Finland; 3 University of Eastern Finland, Department of Forensic Psychiatry, Niuvanniemi Hospital, Kuopio, Finland; 4 Kuopio Research Centre of Geriatric Care, University of Eastern Finland, Kuopio, Finland; 5 School of Pharmacy, University of Eastern Finland, Kuopio, Finland; 6 Research Centre for Comparative Effectiveness and Patient Safety (RECEPS), University of Eastern Finland, Kuopio, Finland; 7 Kuopio University Hospital, Psychiatry, Kuopio, Finland; Medizinische Universitat Graz, AUSTRIA

## Abstract

**Background:**

In register-based pharmacoepidemiological studies, construction of drug exposure periods from drug purchases is a major methodological challenge. Various methods have been applied but their validity is rarely evaluated. Our objective was to conduct an expert-opinion based evaluation of the correctness of drug use periods produced by different methods.

**Methods:**

Drug use periods were calculated with three fixed methods: time windows, assumption of one Defined Daily Dose (DDD) per day and one tablet per day, and with PRE2DUP that is based on modelling of individual drug purchasing behavior. Expert-opinion based evaluation was conducted with 200 randomly selected purchase histories of warfarin, bisoprolol, simvastatin, risperidone and mirtazapine in the MEDALZ-2005 cohort (28,093 persons with Alzheimer’s disease). Two experts reviewed purchase histories and judged which methods had joined correct purchases and gave correct duration for each of 1000 drug exposure periods.

**Results:**

The evaluated correctness of drug use periods was 70–94% for PRE2DUP, and depending on grace periods and time window lengths 0–73% for tablet methods, 0–41% for DDD methods and 0–11% for time window methods. The highest rate of evaluated correct solutions for each method class were observed for 1 tablet per day with 180 days grace period (TAB_1_180, 43–73%), and 1 DDD per day with 180 days grace period (1–41%). Time window methods produced at maximum only 11% correct solutions. The best performing fixed method TAB_1_180 reached highest correctness for simvastatin 73% (95% CI 65–81%) whereas 89% (95% CI 84–94%) of PRE2DUP periods were judged as correct.

**Conclusions:**

This study shows inaccuracy of fixed methods and the urgent need for new data-driven methods. In the expert-opinion based evaluation, the lowest error rates were observed with data-driven method PRE2DUP.

## Introduction

Duration of drug exposure from electronic prescription drug purchase records need to be calculated in order to evaluate persistence of drug use, time to event or current drug exposure at the time of an outcome (for example, hospitalization). Drug purchases as such do not indicate when continuous drug use started and ended, and thus, duration of drug use is calculated from drug purchases with some method including assumptions or restrictions for drug use. Various methods have been created to calculate drug use periods from drug purchases [1]. Caetano et al. classified methods for calculation of drug use periods in persistence research into five groups, from extremely simple anniversary model (at least one purchase per year) to some more realistic models (such as proportion of days covered) in which each refill has an expected length drawn from, for example purchased amount in Defined Daily Doses (DDDs) [2, 3].

There are four methods classes for construction of drug use periods that have been widely used in drug utilization research. The time window method is based on assumption that a drug purchase will last a fixed amount of days e.g. 90 days regardless of purchased amount (i.e. time window of 90 days). An adaption of time window methods is waiting time distribution where duration of purchase is defined according to the time distribution to next purchase of certain drug or package [4]. The second method class (DDD methods) utilizes purchased amount of DDDs with an assumption of a fixed daily dose. Most often use of 1 DDD per day is assumed as DDD is the typical daily dose used for adults for main indication of the drug [3, 5]. The third method class (tablet methods) assumes a fixed daily dose in tablets, and usually this assumption is one tablet per day. This assumption may hold well for some drugs, for example statins are often used one tablet per day [6]. Often a grace period is added to the drug use duration calculated with DDD and tablet methods to allow some irregularity in purchases without generating gaps in drug use periods [7]. The fourth method class is based on either number of days’ supply [8] or written dosage instructions [9]. These methods use information on how long drug purchase should last according dosage instructions.

A newer class of methods are data-driven methods that calculate individual dose and duration estimates from purchase data [10–12]. PRE2DUP (Prescriptions to Drug Use Periods) method [10] is based on modelling of individual drug purchase pattern and do not include fixed assumptions of drug use, such as a specific dose. The method calculates local dose based on purchases and does reality checks for estimated duration of drug purchases according to package-level information. Another method in this class is COV (longitudinal coverage approximation of the applied dose) method which was recently used by Meid et al. [11]. In the COV method, average dose is calculated over the whole previous history preceding the drug purchase to get estimated duration for that purchase.

All these above mentioned methods test if the estimated time on drug reaches next purchase and if so join these purchases to one drug use period. This is done recursively to all purchases of each persons’ drug purchases.

The objective of our study was to conduct expert-opinion based evaluation of the correctness of drug use periods produced by different method classes for selected drugs.

## Materials and methods

### Data source

Data from the Medication use and Alzheimer’s disease (MEDALZ-2005) cohort including all community-dwelling persons with a verified diagnosis of Alzheimer’s disease residing in Finland on 31 December 2005 (n = 28,093) was utilized [13]. Persons with Alzheimer’s disease have been identified from Special Reimbursement register which includes data on entitlement to special reimbursement due to chronic diseases diagnosed by a physician. Diagnoses and data collection are described in more detail by Tolppanen et al. [13].

Data of MEDALZ-2005 cohort has been linked to several nationwide registers including Prescription register, Special Reimbursement register, Hospital Discharge register and register of Care at Social Institutions [13]. Prescription register data includes purchases of reimbursed drugs from all pharmacies in Finland. Data on each purchase includes details of the drug (amount in DDDs, package size, number of packages, strength, and dosage form), date of purchase and costs. For this cohort, altogether 6,115,724 drug purchases were recorded between January 1, 1995 and December 31, 2009 and included in this study. In Finland, drugs may be dispensed for a maximum of three months’ treatment period at a time. The Prescription Register does not cover non-reimbursable drugs, over-the-counter drugs or drugs used in hospitals and public nursing homes. Researchers received de-identified data and no ethics committee approval was required.

### Description of methods included in the evaluation

We evaluated drug use periods calculated with three fixed methods, namely time window method, DDD method, and tablet method, and with PRE2DUP which is a method based on modelling of individual drug use patterns [10]. PRE2DUP represents data-driven method class in this evaluation. In Finland nor days’ supply or written dosage instructions are available in registers and thus methods based on this information could not be evaluated.

Time window method uses predefined time window lengths such as 90 days [14–16] or 180 days [17]. If the following purchase falls into the time window the next purchase belongs to the same drug use period as previous. The method does not take account on purchased amount or package size and does not consider drug use patterns.

DDD method calculates drug use periods assuming a fixed dose in DDDs, usually one DDD per day [18]. The method calculates how many days a purchase lasts with the assumed fixed dose and joins current purchase to the next if the number of days is enough to reach the next purchase. The method is often used with a grace period, which is a fixed number of days that is added to the calculated duration of drug use to allow some irregularity [19, 20]. A grace period may also be added as number of days according to purchased DDDs [21] or combining fixed and proportional grace periods [22].

Tablet methods calculate how many days the purchased amount of tablets will last with a predefined fixed dose which most commonly is one tablet per day. Similar to DDD methods, a grace period is usually added to this model [23–25]

PRE2DUP method is based on individual purchase histories and it uses local dose calculation produced by sliding averages of DDDs [10]. The method also uses package information and for single purchases, adopts the expected drug use duration from users of the same package in the target population. It also tests possible stockpiling and allows personal dosages, dose changes and is independent of drug form (i.e. tablets, injections, inhalations etc.). For stockpiling, PRE2DUP follow the purchasing behavior and tests if the locally calculated dose estimate drops temporarily implying a possible stockpiling event and use of stock. The package information is used to guide method to more realistic drug use patterns including information on if tablets are dividable or not.

### Evaluation of methods

We evaluated drug use periods produced by different methods by expert opinion of two independent reviewers (HT and MK), both having expertise in clinical pharmacy. We evaluated warfarin (Anatomical Therapeutic Chemical code [ATC] B01AA03), bisoprolol (C07AB07), simvastatin (C10AA01), risperidone (N05AX08) and mirtazapine (N06AX11) purchases. The drug use periods were calculated with 13 different methods: time windows 90, 180, 360 days (WIN_90 … WIN_360); 1 DDD per day and grace periods 30, 90, 180 days (DDD_1_30 … DDD_1_180); 1 DDD and 50% proportional grace period which corresponds to 2/3 DDDs per day dose, and the grace period was included in the last purchase (DDD_066_0); 1 tablet per day and grace periods 0, 30, 90, 180 days (TAB_1_0 … TAB_1_180); 1 tablet per day with 50% proportional grace period, which corresponds to 2/3 tablets per day dose, and the grace period was included in the last purchase (TAB_066_0 in tables,) and PRE2DUP. When calculating whether the purchased amount was enough to last to the next purchase with different methods, time spent in hospital between two purchases was removed from the refill time as patients do not use their own drugs during hospital stays. Grace periods were not included in the duration of the last purchase of each drug use period (except for 50% proportional grace models).

Methods were evaluated in two separate sets: one including DDD and time window methods and PRE2DUP, and another including tablet methods with PRE2DUP. These sets included different examples (100 randomly selected purchases for each of the five drugs) because only histories having tablet information (only tablet formulations) for all purchases could be used when evaluating tablet methods. Reviewers reviewed all purchases of the person for that drug during the follow-up to decide correct formation of drug use period containing this selected purchase (when drug use period started and what purchases should be joined together). The information extracted from purchases were purchase dates, number of packages and which package was purchased, amount of DDDs purchased and days in hospital between this and the following purchase if next purchase existed. The evaluation was based on reviewer’s experience on drug use and purchasing patterns. We randomized and blinded the order of the methods for each evaluation (person and drug) to avoid bias. The evaluation included two steps;

1. Determination if drug use period including the selected drug purchase contained the correct drug purchases based on the purchase history of the person. This drug use period was evaluated as correct, incorrect (extra or missing purchases) or non-solvable. The evaluation continued only for those drug use periods that included the correct purchases.
2. We further evaluated if the last purchase of the drug use period had correct duration based on person’s drug purchasing behavior, possible hospitalizations and purchased amount in the last purchase. Reviewer assigned an assumed duration for the last purchase and allowed ±30% variation from this (error marginal).

In this study, “correctness” refers to whether drug use periods included correct purchases and whether the estimated duration was correct according to expert-opinion based evaluation. We categorized expert-opinion based correctness of drug use periods into five groups:

- correct purchases and correct duration (short as completely correct, the main correctness measure),
- correct purchases but false duration (short as correct but false duration, contains correct purchases but duration of the last purchase of the period is not within error marginal),
- correct purchases but non-solvable duration (contains correct purchases but duration of the last purchase of the period cannot be solved because of irregular drug use pattern),
- wrong purchases (extra and/or missing purchases)
- non-solvable (purchase history difficult to evaluate).

The main correctness measure in our study was percentage of completely correct solutions according to expert-opinion. The evaluation was based on reviewer’s experience on drug purchasing patterns and thus, no fixed dose assumptions were used in the evaluation.

Inter-rater reliability was calculated with Cohen’s Kappa. Statistics were calculated with R 3.01 (www.R-project.org). Methods were implemented with dBase 9 (dBase LLC, Binghampton, NY).

## Results

### Fixed time methods

**Fig 1:**
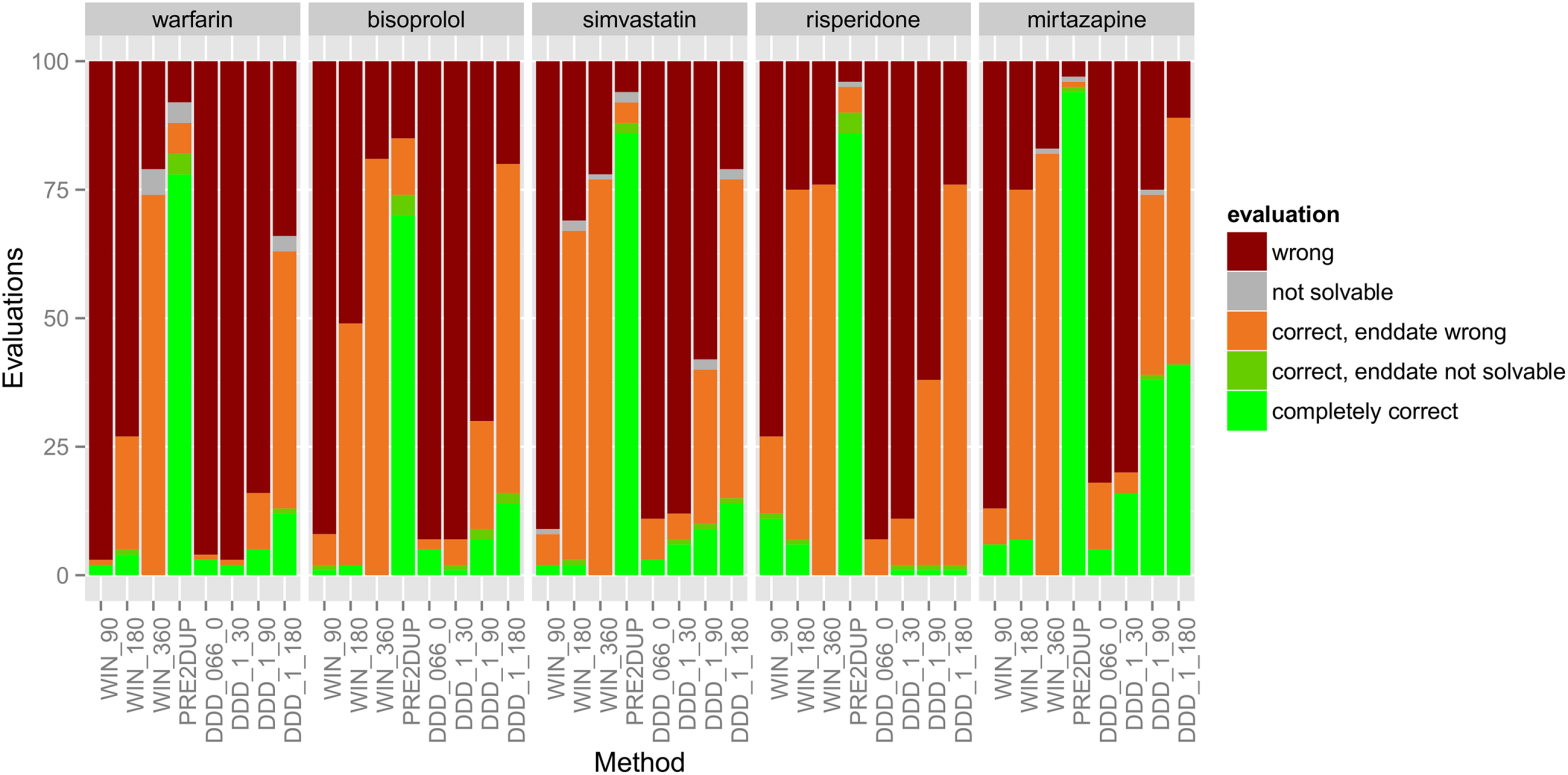
Evaluation results of 100 drug use periods produced by DDD methods along with PRE2DUP for five drugs. Abbreviations explained in text.

Time window methods gave completely correct drug use periods for 0–11% of the evaluated cases (ie. evaluated drug use periods) (Fig 1). Of these methods, time window method of 90 days reached the highest expert-opinion evaluated correctness for risperidone (11%) and 1–11% when considering all drugs.

### DDD methods

DDD methods gave completely correct drug use periods for 0–41% of the cases, and the best performance was noticed for 1 DDD per day with 180 days grace period (DDD_1_180). DDD_1_180 produced completely correct solutions in 1–41% of the cases for different drugs. In overall, the highest expert-opinion evaluated correctness rates for DDD methods were noticed for mirtazapine (41%). In the evaluation set including time windows, DDD methods and PRE2DUP, the average rate of completely correct solutions over all drugs was best with PRE2DUP (83%), DDD_1_180 was second best (16%), and the rest of methods stayed under 10% correctness level. For risperidone, DDD methods performed very poorly as proportion of completely correct drug use periods varied between 0% (DDD_066_0) and 1% (other DDD methods).

### Tablet methods

**Fig 2:**
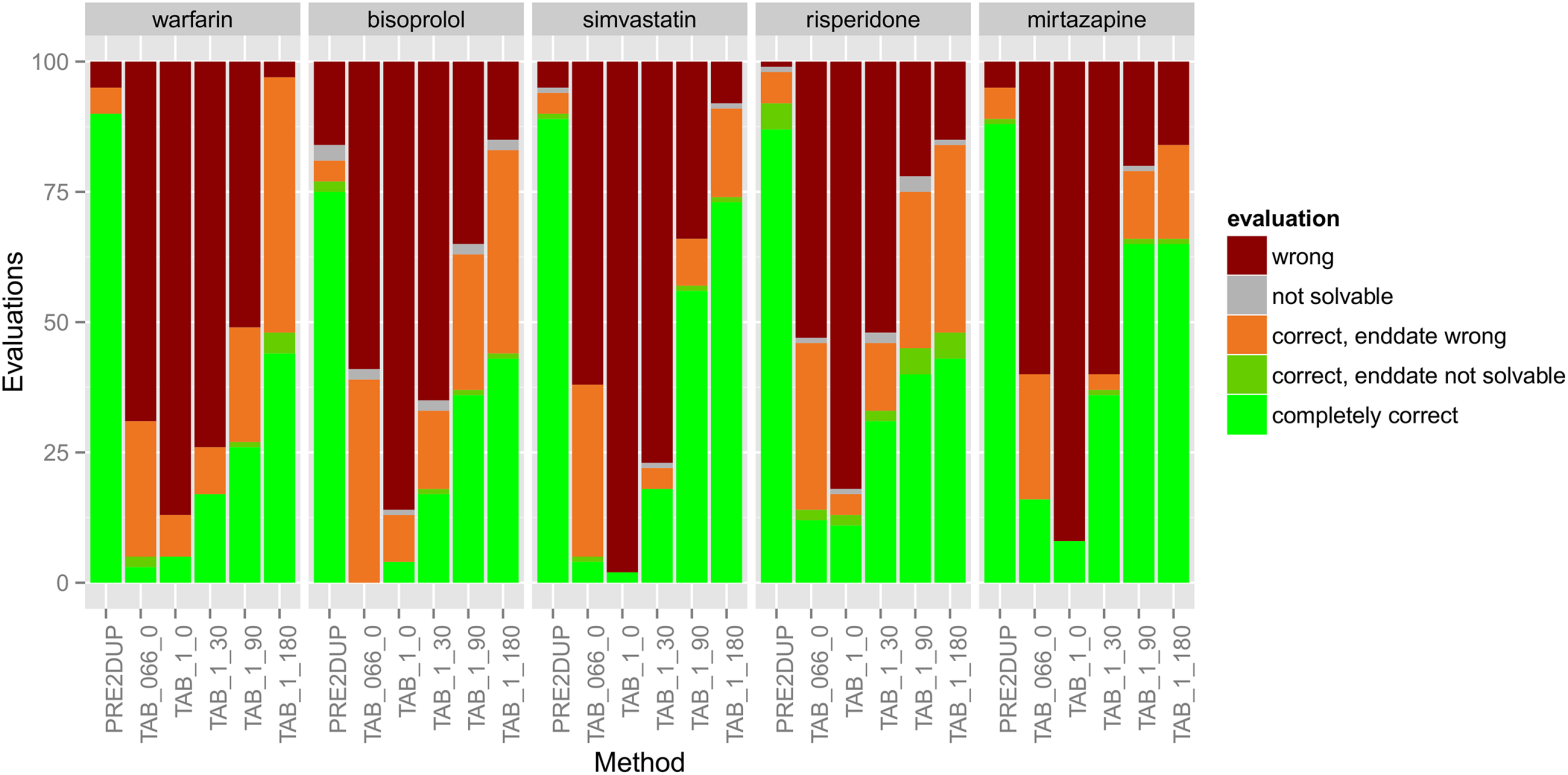
Evaluation results of 100 drug use periods produced by tablet methods along with PRE2DUP for five drugs. Abbreviations explained in text.

Tablet methods produced completely correct drug use periods for 0–73% of the cases (Fig 2). The highest expert-opinion based correctness was reached with 1 tablet per day with 180 days grace period (TAB_1_180) which resulted in 43% to 73% of correctness. Tablet method without grace period showed the worst performance (correctness between 2% and 8%). Overall for tablet methods, completely correct solutions were less common for risperidone (11% to 43%), bisoprolol (0% to 43%) and warfarin (3% to 44%) whereas higher correctness were reached for simvastatin (2% to 73%) and mirtazapine (8% to 65%).

### PRE2DUP method

For PRE2DUP, percentage of completely correct solutions varied between 70% (95% CI 62–79%, bisoprolol) and 94% (95% CI 91–99, mirtazapine) in DDD evaluations (Fig 1) and between 75% (95% CI 68–84%, bisoprolol) and 90% (95% CI 85–95, warfarin) in tablet evaluations. No other method reached 80% correctness in expert-opinion based evaluation.

### Best results for different methods

The best performing fixed method TAB_1_180 reached highest correctness 73% (95% CI 65–81%) for simvastatin for which PRE2DUP reached 89% (95% CI 84–94%) completely correct solutions. The lowest correctness for PRE2DUP was 70%, (95% CI 62–79) for bisoprolol which was still significantly higher than the second best method TAB_1_180 (43%, 95% Cl 33–53). PRE2DUP had 86% average correctness rate over five drugs, and the corresponding rate for TAB_1_180 was 54%, and 45% for TAB_1_90 which had highest rates of all fixed methods. Other tablet methods had average correctness rates between 6% and 24%.

### Joining of purchases correctly

When considering completely correct, “correct purchases but false duration” and “correct purchases but non-solvable duration” together, time window methods yielded at best to 82% correct purchases (time window of 360 days for mirtazapine), DDD methods at best to 89% correct purchases (DDD_1_180 for mirtazapine), and tablet methods at best to 97% correct purchases (TAB_1_180 for warfarin). Calculated this way, PRE2DUP gave correct purchases between 81% and 98% for different drugs. The amount of correct purchases joined but wrong duration after last purchase was remarkably high for DDD_1_180 (48–74%), WIN_180 (22%– 68%) and WIN_360 (74%–82%).

### Error rates

The amount of wrong purchases joined varied from 17% to 24% for WIN_360, from 11% to 34% for DDD_1_180 and from 3% to 16% for TAB_1_180 method (representing the lowest rates of each method class). The highest error rates were 73–97% for time window 90 days, 82–96% for 1 DDD per day with 50% grace period and 82–98% for one tablet per day without grace period. For PRE2DUP amount of erroneous results varied between 1% and 16%.

### Reviewer’s concordance

The agreement between two reviewers (HT and MK) was high and they rated correctness (with five possible options) similarly in 93–99% of the evaluated drug use periods produced by different methods Table 1. Corresponding Kappa values were between 0.88 and 0.98.

**Table 1:** Percentage of equal evaluations by two reviewers in two evaluation sets, referred as DDD set and TABLET set.

Drug (ATC code)	DDD set %	TABLET set %
Warfarin (B01AA03)	99	94
Bisoprolol (C07AB07)	96	96
Simvastatin (C10AA01)	97	99
Risperidone (N05AX08)	97	95
Mirtazapine (N06AX11)	96	93

## Discussion

### Main findings

In expert-opinion based evaluation, data-driven PRE2DUP performed significantly better than fixed methods for all drugs. Our results demonstrate that choice of method is crucially important for producing correct duration of drug use calculated from prescription drug purchases.

### Performance of PRE2DUP

PRE2DUP yielded fairly high proportion of completely correct drug use periods (70–94%) and had the best performance of the tested methods in this expert-opinion based evaluation. PRE2DUP gained highly correct results both with regular, long-term drugs such as simvastatin and with drugs which are used with varying doses (warfarin, risperidone). The poorest performance was observed with bisoprolol with correctness 70% and 75%, compared with 94% correctness in mirtazapine use periods. The amount of wrong purchases joined was highest (16%) for bisoprolol. This was caused by the fact that package wise parameters were not included for bisoprolol while they were available for all other evaluated drugs. This demonstrates that for a regularly used drug, error rate of PRE2DUP can be substantially reduced with package parametrization. The largest differences with PRE2DUP and best performing tablet methods were observed with drugs that have high within- and between-individual variation in dose, namely warfarin and risperidone. For these drugs, data-driven PRE2DUP had significantly higher correctness than any fixed method. These results are in concordance to agreement with interview and PRE2DUP modelling results among older persons [26].

### Performance of fixed methods

TAB_1_180 was the best performing method for most drugs after PRE2DUP. However, there was large variation in the expert-opinion based correctness produced by the method; it produced 73% of completely correct results for simvastatin but only 43% for risperidone. This highlights the fact that none of the fixed methods is universally usable for all drugs, drug use patterns and drug user populations [27]. The performance of tablet methods was improved with longer grace periods when we did not add grace periods to the end of drug use period. The best performance of DDD methods was seen for mirtazapine (5% to 41% correctness) but also for that drug, tablet methods performed better (8% to 65% correctness). The choice of method to calculate drug use periods depends on population of interest and how drugs are used (drug purchasing behavior). Predefined dose assumptions should always be tested in the population of interest, for example by calculating average dose of the drug based on refill time lengths in the population. One should also consider if all products of the drug are used in the same manner, for example dosage forms may cause alterations.

Tablet methods produced higher correctness compared with DDD and time window methods which most commonly did not reach correct duration for the last purchase of the drug use period. This was most prominent for simvastatin which is most commonly used with one tablet per day dose and long grace periods of 90 and 180 days allow tolerance for irregularity of purchases. In our evaluation examples, we did not add grace period to the duration of the last purchase and thus, no stock or deviance from assumed dosage were added to or subtracted from the last purchase. This was seen in bisoprolol results as it may be used two times a day to avoid intolerably high single doses. One tablet per day assumption will result in wrong end date for a user with dose of 2 tablets per day. Percentage of “correct purchases but wrong duration” was higher for bisoprolol than for simvastatin in tablet methods. Long grace periods allow lower doses than defined in the assumption (for example, 0.5 tablets per day) but when the last purchase is calculated as one tablet per day without adding grace period this may lead to wrong end date for users of 0.5 tablets or DDDs per day (ie. purchase should last two times longer than calculated with fixed one tablet per day assumption).

### Study population

Our study population with advanced age and Alzheimer’s disease later during the follow-up used many drugs with dose less than 1 DDD per day [27, 28] and thus, methods with assumption of 1 DDD per day use led to poor results for DDD methods with short grace periods. However, this problem concerns all populations, not only ours. Some drugs are rarely used 1 DDD per day even in the general adult population [6]. The reason for this is that DDD value for some drugs (for example, simvastatin) is set between two most commonly used doses and almost no one uses 1 DDD per day in any population. The average dose used by the study population is not known beforehand, and if fixed assumptions are used the doses should always be calculated from data for each drug or drug package.

### Implications for future research

The results of our study point out that fixed assumptions of drug use for the whole population may produce drug use estimates that are mostly incorrect. Incorrect exposure durations may be hazardous in studies assessing the relationship between drug exposure and an outcome event and impose serious bias [29]. Previous studies have also reported that varying definitions for duration of a dispensing may markedly lengthen exposure duration when studying selective serotonin reuptake inhibitor use [30]. Our results are in line with Meid et al. where data-driven COV (estimation of drug coverage) method produced better results than fixed methods assuming use with fixed dosage [11].

To overcome fixed assumptions for all drugs and serious problems related to those, drug use patterns can be calculated separately for each drug substance or package in the whole population [4]. The next step is to calculate individual drug use time, either with dosage instructions [9] or with average dose calculated from individual purchase histories either in local (PRE2DUP) or longitudinal (COV) way [10,11]. The duration estimates for single purchases without information on personal history can be drawn from drug use patterns of the study population [4, 10, 11]. These patterns can be stratified according age, sex or other measures affecting drug use patterns to produce better duration estimates. One limitation is that data-driven methods using parameters calculated from purchase data may use information over the whole study period and thus, purchases after a time point of interest can affect the results. This can introduce bias if for example outcome event changes drug purchasing behavior.

### Strengths and limitations

Applicability of our results relate to the features of our study population and healthcare setting. These results represent older Finnish persons with Alzheimer disease and their specific drug use patterns in exemplary drug treatments with concomitant use of several drugs, high number of comorbidities and hospitalizations. Older persons are frequent drug users and represent the ultimate challenge for drug use modelling. Thus, a method that performs well in this population is also likely to perform well in populations with simpler drug use patterns and fewer comorbidities. However, different healthcare systems have different regulations for dispensing and reimbursement which has to be taken into account. For this reason, method comparison is fairly applicable to countries with similar dispensing systems and regulations, especially in Nordic countries. Two independent reviewers produced very similar results. This implies that this method comparison was fairly reliable. However, a limitation of this study is that the correctness was based on expert opinions and we do not know “true” drug use patterns i.e. whether the dispensed drugs were actually taken, when they were taken and for how long the drug use actually continued.

For comparisons, we selected fixed methods that were used in previous studies. However, our limitation is that all possible methods could not be included and some important methods may be lacking. Thus, future studies with different methods and approaches are needed. The evaluation was made for five drugs that were commonly used in the population and represent different drug use patterns according to our experience. With different drugs, the evaluation results would evidently be different for certain methods. As expert-opinion based evaluation is very time consuming we were not able to conduct it for a larger selection of drugs.

## Conclusions

The results of our study point out that fixed assumptions of drug use for whole population may produce drug use estimates that are mostly incorrect. Data-driven PRE2DUP showed highest expert-opinion based correctness of drug use periods over all methods studied. Fixed methods in general performed only moderately even in best cases. Time window methods should be used with great caution for calculation of drug use periods although they may join purchases correctly. Also the performance of DDD methods was moderate. Tablet methods may produce fairly good results for certain drug classes and patient groups, but misclassifications may be enriched in selected, not random subpopulations and thus, bias the results. All fixed methods perform badly when calculating duration for the last purchase and fixed assumption should not be applied to the last purchase. Data-driven methods like PRE2DUP overcome these limitations and have superior performance producing drug use periods with correct purchases and correct duration.

More research is needed to compare different methods and the deviance of results. As different methods produce different drug exposure times, the impact of this on research results should be investigated. This evaluation should be applied to risk of adverse outcomes associated with current drug exposure, as well as with cumulative or long-term use.
